# High-throughput high-volume nuclear imaging for preclinical in vivo compound screening^§^

**DOI:** 10.1186/s13550-017-0281-4

**Published:** 2017-04-07

**Authors:** Sven Macholl, Ciara M. Finucane, Jacob Hesterman, Stephen J. Mather, Rachel Pauplis, Deirdre Scully, Jane K. Sosabowski, Erwan Jouannot

**Affiliations:** 1 inviCRO Ltd, Charterhouse Square, London, EC1M 6BQ UK; 2 inviCRO, LLC, 27 Dry Dock Avenue, 7th Floor West, Boston, MA 02210 USA; 3 Centre for Molecular Oncology, Barts Cancer Institute, Queen Mary University of London, Charterhouse Square, London, EC1M 6BQ UK; 4 Sanofi Aventis Recherche Développement, 1, Avenue Pierre Brossolette, 91380 Chilly-Mazarin, France

**Keywords:** Nuclear imaging, Automation, Image analysis, Compound screening, Biodistribution, Pharmacokinetics

## Abstract

**Background:**

Preclinical single-photon emission computed tomography (SPECT)/CT imaging studies are hampered by low throughput, hence are found typically within small volume feasibility studies. Here, imaging and image analysis procedures are presented that allow profiling of a large volume of radiolabelled compounds within a reasonably short total study time. Particular emphasis was put on quality control (QC) and on fast and unbiased image analysis.

**Methods:**

2–3 His-tagged proteins were simultaneously radiolabelled by ^99m^Tc-tricarbonyl methodology and injected intravenously (20 nmol/kg; 100 MBq; *n* = 3) into patient-derived xenograft (PDX) mouse models. Whole-body SPECT/CT images of 3 mice simultaneously were acquired 1, 4, and 24 h post-injection, extended to 48 h and/or by 0–2 h dynamic SPECT for pre-selected compounds. Organ uptake was quantified by automated multi-atlas and manual segmentations. Data were plotted automatically, quality controlled and stored on a collaborative image management platform. Ex vivo uptake data were collected semi-automatically and analysis performed as for imaging data.

**Results:**

>500 single animal SPECT images were acquired for 25 proteins over 5 weeks, eventually generating >3500 ROI and >1000 items of tissue data. SPECT/CT images clearly visualized uptake in tumour and other tissues even at 48 h post-injection. Intersubject uptake variability was typically 13% (coefficient of variation, COV). Imaging results correlated well with ex vivo data.

**Conclusions:**

The large data set of tumour, background and systemic uptake/clearance data from 75 mice for 25 compounds allows identification of compounds of interest. The number of animals required was reduced considerably by longitudinal imaging compared to dissection experiments. All experimental work and analyses were accomplished within 3 months expected to be compatible with drug development programmes. QC along all workflow steps, blinding of the imaging contract research organization to compound properties and automation provide confidence in the data set. Additional ex vivo data were useful as a control but could be omitted from future studies in the same centre. For even larger compound libraries, radiolabelling could be expedited and the number of imaging time points adapted to increase weekly throughput. Multi-atlas segmentation could be expanded via SPECT/MRI; however, this would require an MRI-compatible mouse hotel. Finally, analysis of nuclear images of radiopharmaceuticals in clinical trials may benefit from the automated analysis procedures developed.

**Electronic supplementary material:**

The online version of this article (doi:10.1186/s13550-017-0281-4) contains supplementary material, which is available to authorized users.

## Background

In vivo tissue biodistribution of administered drug or imaging agent candidates is fundamental for their pharmacological assessment [2], and its evaluation is required for the translation from in vitro research to the clinic, see e.g. [3] on radiopharmaceuticals [4]. Medicinal chemists also study compound biodistribution as an in vivo screen following in vitro tests (which e.g. confirm target binding). The in vivo assessment focusses on pharmacokinetics, here, bioavailability (concentration in plasma), delivery to and retention at the target and off-targets, and systemic clearance over time. This assessment may also include study of metabolism (DMPK). Experimental methods can be distinguished [2, 5] e.g. between ex vivo (e.g. autoradiography [6]) and in vivo, and between label-free (e.g. Mass Spectrometry Imaging [7]) and labelled [8]. All these methods have their merits and drawbacks, but generally, they are all low in throughput [9]. That low throughput makes these tools unattractive for the screening of larger combinatorial libraries of compounds. One solution is replacement by in silico or in vitro screening, but results of current techniques may not be reliable enough [10]. In vivo optical imaging may be chosen to increase throughput [11], but this comes at the cost of limited spatial resolution and limited quantitation [12]. In this study, radionuclear imaging has been chosen which provides quantitative data [13] and for which radiolabelling automation is possible [14].

Another important consideration is the large number of animals required for an ex vivo study to cover a sufficient number of time points and to yield statistical significance [15]. Humane animal research demands observing the replacement, reduction and refinement (3Rs) [16] which includes reducing the number of animals. This reduction may be achieved by non-invasive imaging allowing repeated, longitudinal probing in each animal post-compound administration. As a result, the reduction factor equals the number of time points. For example, the NCRI guidelines recommend to use 2 to 3 animals at 5 to 8 time points for a pharmacokinetic study [17]. This corresponds to 10 to 24 animals in total with an ex vivo method compared to 2 to 3 animals with longitudinal in vivo imaging.

One high-throughput ^18^F positron emission tomography (PET) study has been reported [18] where 42 compounds were imaged within 11 days, at up to 3 h pi. The impressive throughput did not include time for image analysis which in that study was performed manually and thus would prolong total study time considerably. Therefore, besides employing longitudinal, multi-animal imaging and efficient radiolabelling, overall study throughput was increased in our study by automated image analysis. Furthermore, quality control was implemented at all relevant steps within the whole process to ensure high data quality.

In this study, workflows, methods and software have been developed to allow efficient high-volume compound screening of 25 proteins by SPECT/CT of a tumour xenograft mouse model. The main interest of this study was to explore pharmacokinetics and tumour uptake in vivo in a large panel of compounds with different biologic properties (e.g. size, charge, and format). Specific goals were to (1) quantify the test item uptake to the tumour in a patient-derived xenograph (PDX) mouse model, (2) quantify systemic biodistribution over up to 2 days post-injection (pi), and (3) confirm last time point imaging data by ex vivo analysis. The ultimate aim for the medicinal chemists and pharmacologists was guidance for further drug design efforts. These efforts, including the preceding, mandatory in vitro characterization of this compound library, target validation and animal model validation, will be described elsewhere by Sanofi affiliated researchers.

## Methods

### Test compounds

All 25 proteins were designed and synthesized including a hexahistidine sequence (His-tag) serving two purposes: improving compound purification and enabling radiolabelling by the ^99m^Tc-tricarbonyl methodology. These His-tagged proteins had been used in preceding in vitro target binding affinity studies. Radiochemistry, imaging and image analysis staff were blinded to compound properties except for the individual molecular weights (14 to 86 kDa).

### Radiolabelling

Small aliquots of all 25 proteins were radiolabelled first for testing, following the recommendations on ^99m^Tc-tricarbonyl labelling given in [19]. Subsequently, larger aliquots were radiolabelled for imaging. On each imaging start day, usually 2 or 3 proteins (1.6 to 1.9 nmol of each) were radiolabelled using 1 Isolink kit (Paul Scherrer Institute, Switzerland) and 2.5 GBq ^99m^Tc-pertechnetate. After 2 h incubation (3 h for 5 compounds to yield sufficient labelling efficiency), the product was purified on a NAP-5 column (GE Healthcare). Aliquots of the product solution were used for instant thin layer chromatography (ITLC) and for size exclusion high-performance liquid chromatography (HPLC). The remainder of typically 500 to 600 μg was split into 3 portions for injections. See Additional file 1 for further details.

### Reducing renal uptake

To reduce renal uptake of test items, aqueous L-lysine monohydrochloride (Sigma-Aldrich) solution (pH 7.3) mixed with gelofusine (Gelaspan, B. Braun Melsungen, Germany) was administered [20, 21] as intravenous (iv) bolus at 4 mL/kg 30 min before radiotracer injection. Mass doses were 0.1 g/kg gelofusine and 1 g/kg L-lysine.

### Animal model

All animal procedures were approved by the Animal Welfare and Ethical Review Body at Queen Mary University of London and by the UK Home Office in accordance with EU Directive 2010/63/EU. Female Fox Chase SCID mice (Charles River, UK) were 6–8 weeks old on arrival. A first batch of 8 mice was shaved on the lower back (1 cm^2^), local anaesthetic cream (lidocaine and prilocaine) applied, and a≈10 μL fragment of primary human colon adenocarcinoma tissue (from patient CR-IGR-034P, tumour collection CReMEC, Oncodesign, [22]) injected subcutaneously in an intrascapular position by trocar under isoflurane anaesthesia. The wound was closed with spray plaster, and animals were monitored more frequently for 2 days. Once tumours had grown to 8–10 mm diameter, one animal was sacrificed each week, the tumour excised and cut into ≈10 μL pieces for direct passaging into a batch of 20–24 animals (5 batches in total), following the same inoculation procedure as above. All animals underwent regular body weight and tumour size measurements and examinations for any signs of abnormalities.

### Imaging

For each compound, three mice bearing a tumour of ≈0.1 to 0.4 mL were selected randomly, weighed and bolus iv injected with lysine/gelofusine and the test item (5 mL/kg, 20 nmol/kg protein, ≈100 MBq) into each of the lateral tail veins. The delivered test item doses were calculated from the syringe weights and radioactivity measurements before and after injections. All three mice were imaged together on a multi-mouse bed (Minerve, France) equipped with anaesthesia system (≈2% isoflurane in 1.5 L/min medical oxygen) and warm air ventilation, on a NanoSPECT/CT camera (Bioscan Inc., USA).

**Fig. 1:**
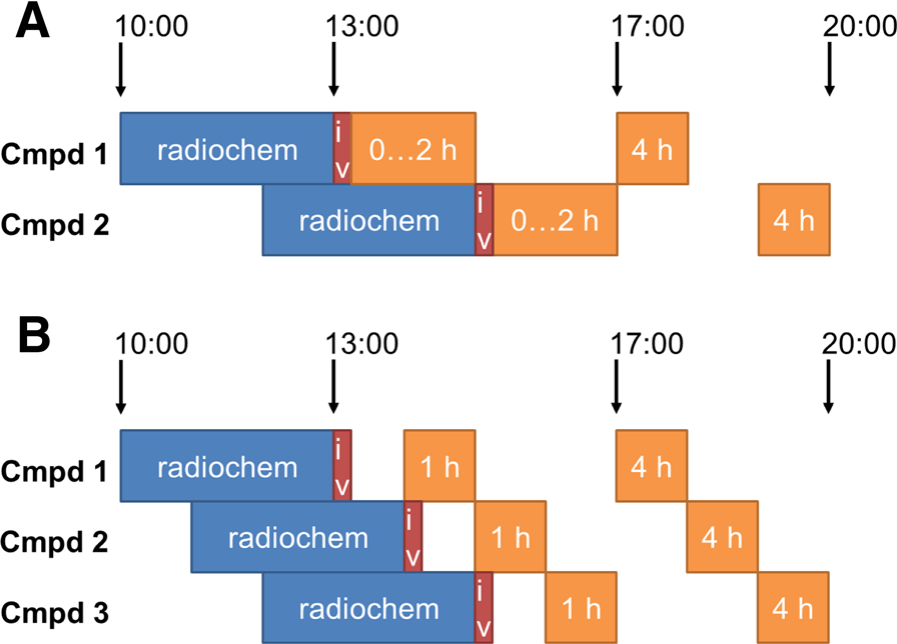
Examples for scheduling radiolabelling, injections and in vivo imaging up to 5 h pi (initial part of the whole schedule). **a** Two compounds with initial dynamic imaging over 2 h. **b** Three compounds with SPECT acquisition starting at 1 h pi

The first SPECT image was acquired either at 1 h pi for 50 min (radiotracer injection and first hour thereafter without anaesthesia) or as a series of 10 SPECT images between 10 and 110 min pi (radiotracer injected under anaesthesia which continued for the dynamic imaging scan), followed by a CT scan (10 min, 240 projections with 1 s exposure to 55 kVp X-rays). Further SPECT/CT images were acquired at 4 and 24 h pi and for some compounds at 48 h pi (always 50 min SPECT). This schedule allowed for staggering of 2 or 3 groups of animals (i.e. compounds) at a time, see Fig. 1.

SPECT and CT images were reconstructed with an iterative algorithm (HiSPECT, Scivis GmbH, Germany) and with exact cone beam Filtered Back Projection (VivoQuant, inviCRO LLC, USA), respectively.

### Image analysis

All image processing was performed in VivoQuant 2.0 (inviCRO) and iPACS (inviCRO). Preprocessing included SPECT/CT coregistration at a voxel size of (0.4 mm)^3^ and splitting into individual mouse images. An iPACS script facilitated entering injection doses and body weights simultaneously for automatic conversion of image data into absolute radioactivity, percent injected dose (ID) or standardized uptake value (SUV). A VivoQuant script generated and saved rotating maximum intensity projection (MIP) movies and single-slice images in all three orientations centred on the tumour ROI with a chosen colour scale (here 0.2 to 20% ID/mL).

Tissue uptake data from SPECT images were generated via multi-atlas segmentation: a reference library of ROIs is built in usually 10 to 20 animals to create an ROI atlas, which is co-registered to SPECT/CT images. See Additional file 1 for further details.

Data of all image ROIs (volume, % ID, % ID/mL, SUV) were saved, then custom plotted via MATLAB script, e.g. ordered by group (i.e. compound) or by ROI class (i.e. organ, tissue).

### γ-Counting and analysis

Animals were dissected after imaging. Tissue sample weights were transferred by push-button from the balance into a spreadsheet template. Decay-corrected counts-per-minute data of the daily batch of tissue samples (≈100 for 2 compounds) were measured on a γ-counter LKB Wallac 1282 Compugamma within 2 h. All accumulated data of the whole study were then imported into MATLAB by a custom-written script linking data points from different sources via compound and mouse identifiers. Uptake data (% ID, % ID/g and SUV) were tabulated and plotted. Further details including tissue-specific calculations are described in the Additional file 1.

### Quality control procedures and calibrations

Radiolabelling quality control (QC) included (1) ITLC and HPLC of the product to confirm sufficient radiochemical purity (>95%), and (2) measurement of the radioactivity concentration for the preparation of individual radioactivity doses (target≈100 MBq) at a volume dose of ≈0.2 mL.

Mice were monitored daily to control health status, and mice were enrolled into the study only in absence of any abnormalities. QC of tumour volume estimates by calliper measurements was by SPECT/CT and weighing excised tumours (target range 0.1 to 0.4 mL).

Radiotracer injection quality was checked by SPECT. Both the SPECT and CT scanner units passed all manufacturer recommended QC procedures checked before and after the study. Directly after image acquisition and reconstruction, images were inspected to rule out technical issues like motion artefacts or poor injections. QC after image analysis included (1) inspection of all MIP movies and single slice images to confirm proper processing (e.g. coregistration and ROI placements) and image labelling by subject identifiers (comparing all images per mouse) and (2) automated detection of potential outliers in the uptake data.

Ex vivo analysis included automated QC checks on γ-counting rates falling into the linear range of the detector and being well above background, and detection of potential outliers in the tissue weight and uptake data. Finally, all plots were inspected visually.

### Statistical analysis and data plotting

Basic descriptive statistical calculations were performed in MATLAB R2015b (The MathWorks) and Excel 2013 (Microsoft). Data are reported as mean ± standard deviation (SD) if not noted otherwise. Intersubject variability was expressed as % COV (coefficient of variation) for each group of three mice, then taking the median for each uptake measure over all time points, compounds and organs. Details and all organ-specific data are given in the Additional file 1. Bland-Altman analysis and plotting were conducted in Prism 5 (GraphPad Software). All other plots were prepared in MATLAB.

## Results

### Radiolabelling

The preparations for imaging had the following study averages: radiochemical purity = (98.0 ± 1.6) %, activity = (331 ± 82) MBq in (539 ± 59) μL for three injections.

### Animal model

The preparations provided an adequate rolling stock of animals with tumour xenografts despite the challenge of an occasionally variable engraftment latency period apparently not uncommon for PDX models [23]. No complications were encountered with animal procedures except for one ulcerating tumour (excluded from study). Median tumour growth duration was 23 days. Unforeseen logistical circumstances required expedition of the study leading to seven compounds screened in the last week. The increased demand in animals was met by accepting some tumours outside the target size range into the study, see abscissa of left panel in Fig. 4. These cases are readily identifiable in the available scatter plots, and no noticeable impact on uptake was found.

### Injections and imaging

The phantom-derived quantification calibration factor obtained before the in vivo study was consistent with historic data for that scanner and with a confirmatory calibration check after the in vivo study (within 4%).

**Fig. 2:**
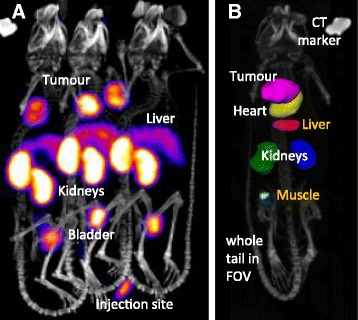
Exemplary images. **a** “Raw” SPECT/CT image of three mice as maximum intensity projection. **b** CT image overlaid with 3D ROI volumes of whole tumour, heart and kidneys, and of small liver and muscle portions

Figure 2a shows an example of a SPECT/CT image of the three mice in the hotel before splitting into individual animals. Excellent image contrast is obtained in the SPECT image for tumours, kidneys and bladders. Typical three-dimensional ROIs are shown in Fig. 2b on a CT image. Eventually >500 single animal SPECT/CT images were acquired and analysed for the 25 test compounds within 5 weeks. All images were made available on the iPACS for manual inspections and archiving. They were also arranged in annotated slides decks with rotating MIP and single-slice image views as SPECT, SPECT/CT and ROI/CT.

**Fig. 3:**
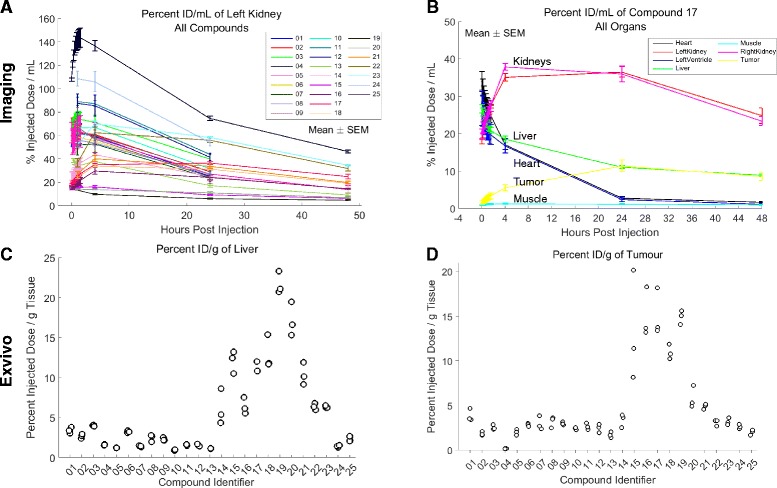
Exemplary plots of imaging data (*top row*, mean and SEM *error bars* for *N* = 3) and ex vivo data (*bottom row*, individual data points). **a** % ID/mL in left kidney ROIs (all compounds, all time points). **b** % ID/mL data of compound 17 (all ROIs, all time points). **c** % ID/g in liver in all animals (≈24 or 48 h pi). **d** % ID/g in tumour in all animals (≈24 or 48 h pi)

Image analysis produced SPECT data of ≈4000 ROIs. Examples for SPECT data plots are provided in Fig. 3a, b. While each plot condenses information from at least 9 SPECT images (three animals, three time points) or even from ≈500 (all animals, all time points as in Fig. 3a), the subdivision into tissues and compounds, and the choice of parameters and various plot types resulted again in an expansion, here to >1000 plots. This was managed by tables of contents in PDF slide decks with a hierarchical folder-structure allowing quick access to any plot of interest.

Intersubject variability for tumour, heart, kidneys and liver was 13% for all considered uptake measures (% ID, % ID/mL, and SUV), ranging from 7% for liver SUV to 19% for heart SUV.

### Ex vivo analysis

Circa 1000 tissue samples were collected, weighed and counted, in typical daily batches of ≈100 or 150 samples. All data analysis was automated and results were available in near real-time. However, often a delay of 1–2 days between sampling and counting was required for very high activity samples to sufficiently reduce γ-counter dead-time. Data of such repeat measurements automatically replaced QC-rejected data of the first counting run.

The final data set was visualized in an automatically generated PDF slide deck of 172 plots, see examples in Fig. 3c, d.

**Fig. 4:**
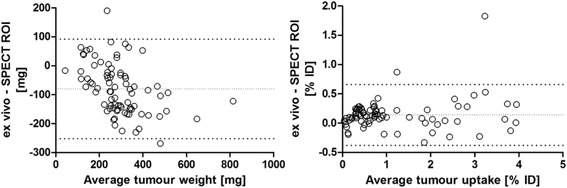
Bland-Altman plots on the difference in tumour data between ex vivo (dissected tissue) and in vivo (SPECT/CT) methods, based on pairs of data from the same animal. *Left panel*, tumour weight (ex vivo) and tumour volume (SPECT ROI) (converted to weight assuming tissue density = 1.0 g/mL); *right panel*, radiotracer uptake in tumour as % ID (ex vivo from gamma counting and in vivo from SPECT ROI). Bias as *dotted line* (at −80 mg and +0.14% ID, respectively), 95% agreement interval as *pair of bold dotted lines*. Values on the *abscissa* are individual tumour averages over the two measurement methods

Biodistribution data from ex vivo dissection and SPECT were compared by Bland-Altman analysis, see Fig. 4 for examples.

## Discussion

### Study results

Preliminary test radiolabelling proved useful in cases where insufficient radiolabelling efficiency was solved by longer incubation. Subsequent radiopreparations for imaging all passed QC.

The only problem encountered with the PDX mouse model was engraftment latency in some animals of the first passage. Despite an initially reduced choice of animals for imaging, significant study delays were avoided. In parallel to regular animal welfare checks, an efficient and frequent stock checking procedure allowed to continuously adapt the schedule for tumour inoculations and for optimal selection of animals for imaging.

This study did not attempt to study the effect of lysine and gelofusine on the biodistribution but rather utilized this as a blanket method to reduce kidney uptake. Control experiments to quantitate the absolute or relative kidney uptake reduction with different radiotracers, e.g. compounds of different molecular weight, were out of scope because they would have doubled the number of experiments. For the presented study, the kidney uptake data have to be interpreted very carefully. However, since renal clearance is downstream of delivery to other tissues of interest (e.g. tumour), no significant effect is expected on these.

The initial study design was based on 13 SPECT images (of the mouse hotel, *N* = 3 mice) per compound starting with dynamic scanning over the first 2 h post-radiotracer injection and ending at 48 h pi. To cut costs and to reduce stress to animals, this imaging schedule was reduced in the final study plan for 18 of the 25 compounds by removing the last time point and/or reducing the initial dynamic scanning to a 1 h “static” image acquisition. Detailed comparisons of compounds with these 2 different first image acquisitions may need to consider the different durations under isoflurane anaesthesia.

### Image analysis

Region of interest definition methods were chosen to strike a balance between precision, consistency and efficiency. Concentration (% ID/g, SUV) estimates allow the use of fixed volume regions. These benefit from generally low anatomical intersubject variability in preclinical studies. Examples are liver and kidneys. For liver with its complex organ shape, a subregional ROI near the organ centre was used under the assumption of homogeneous distribution throughout the organ tissue. Kidneys have a simpler organ shape but can exhibit inhomogeneous SPECT signal distribution. Therefore, a fixed volume encompassing ROI was used.

For subcutaneous tumours with their often inhomogeneous SPECT signal distribution, inconsistent size and shape and more variable location, ROIs were generated manually. Still, for the voxel size and tumour sizes in this study, erosion of or dilation by a single voxel layer from the surface of a 3D ROI changes volume estimates by tens of percents. Therefore, ROI volume estimates were compared to ex vivo tumour weights as QC. In cases of large discrepancy, the tumour ROI was revisited and, if necessary, edited.

Multi-atlas segmentation has proven useful for automated region identification especially in clinical neuro-applications [24–27]. To overcome the limitation of many available software packages designed for clinical brain imaging, this approach has been implemented and employed for full and subregion segmentation of other organs (for whole body distribution and radiation dosimetry) in several non-human species. Data variability—due to factors such as animal positioning, the non-rigidity of non-brain regions, general shape/distribution outliers and fluctuating contrast-to-noise—may occasionally cause segmentation failures, thus requiring strict QC and manual corrections. Nevertheless, we found in timing experiments (not shown here) that multi-atlas segmentation followed by user editing of the automatically generated ROIs still significantly decreases processing time and observer variability compared to segmenting regions manually de novo.

Optimal subject number is a challenging question for preclinical studies. A larger number of mice as normally used in a dissection study allows for better inference towards the mouse population compared to the often reduced number of mice used for imaging. On the one hand, an animal group size *N* > 3 is suggested in some circumstances [15, 28]. On the other hand, longitudinal imaging of a single mouse removes that intersubject variability and may allow for a clearer observation e.g. of radiotracer kinetics [29]. In this study, statistical analysis was utilized but not an overarching driver. Rather, the goal was to achieve sufficient consistency and power in methodology to identify compounds of interest to be evaluated more thoroughly in follow-up experiments and with additional in vitro results at hand. Figure 3a, c shows that indeed relevant, apparent differences between compounds are often much greater than intersubject variability at the chosen group size of three. Ultimately, the choice of the group size represents a compromise between statistical needs for in vivo and ex vivo study design and study aim, logistical implications, cost and ethics.

### Ex vivo

Ex vivo data were acquired to validate the biodistribution results from imaging which was successful as Bland-Altman analysis showed. One observation was, however, that the SPECT tumour ROI volume tended to be significantly larger than the ex vivo tumour weight. This discrepancy is likely due to an overestimation of the tumour volume on the images, and deviation from the assumed 1 g/mL mass density may also play a role. Such an overestimated ROI volume likely captures spillover of the tumour SPECT signal just outside the tumour, an occasional artefact from image reconstruction or smoothing. This conjecture would explain the good match of tumour uptake values (% ID) between ex vivo and in vivo measurements and also explain the observed non-equivalent measures of uptake concentration in tumour (ex vivo % ID/g versus imaging % ID/mL). As discussed above, the ROI drawing protocol can be tailored to generate ROIs that achieve a better match in either % ID (as for tumours in this study) or % ID/g values between ex vivo dissection and in vivo imaging data.

### Throughput

All experiments from animal model setup to final analysis took 3 months. This included time for the animal model setup which could be eliminated in other studies when using naïve animals, or which might take longer for certain disease models.

This study design for large proteins included imaging up to 48 h pi. In hindsight, this time point adds only limited information to that obtained at 24 h and could be omitted in a future study of such compounds, making space in the schedule available for other experiments.

Image analysis of incoming data was done within a day to create updated study plots. A fortunate benefit was that image analysis was performed in a later time zone than data acquisition (GMT→EST), effectively stretching the working day.

γ-counters are more sensitive than SPECT cameras. Therefore, the fairly large injected radioactivity dose for imaging needs to decay considerably to reach the measurement window of a γ-counter. This slows down throughput, but after successful validation as in this study, ex vivo analysis could be omitted altogether from future imaging studies.

High volume imaging generates large amounts of data, but for preclinical SPECT/CT or PET/CT, this is well manageable within a contemporary standard information technology environment.

### Subsequent use of the data

Compound ranking, or identifying compounds fulfilling a set of inclusion criteria, or elimination of poorly performing compounds can be based on algorithms combining several uptake, uptake rate or clearance measures and limits. A separate paper will apply such an algorithm in combination with in vitro and in silico measures. Follow-up in vivo experiments related to pharmacokinetics may be performed on a selected small group of potential lead candidates, e.g. on compound/radiolabel stability in vivo.

## Conclusions

Feasibility and application of high-volume in vivo compound screening by preclinical SPECT/CT and automated analysis have been demonstrated. Excluding the animal model setup, the required time for such a study including a final report is on the order of weeks. Importantly, longitudinal imaging reduced the number of animals three- to fourfold compared to dissection studies, even 12- to 13-fold when considering the dynamic scans in this study.

Specific for this study was the compound type, large proteins, for which (a) a test on intact biological functionality after radiolabelling may be skipped (or replaced by in vitro target binding affinity measurements of the His-tagged but unlabelled compounds as done here), and (b) a “one size fits all” radiolabelling procedure may work.

The whole process proved efficient and robust. High confidence in the data set was achieved by (1) blinding the contract researchers to compound properties, (2) quality control checks at all stages, (3) transparency of raw data and analysis procedures and easy access to all data, and (4) a high automation level in data acquisition and analysis.

Additional ex vivo data validated the image analysis approach. While systematic differences in the output parameters can be observed, these can be corrected for if desired, and they are not expected to affect the relative compound ranking. Future studies employing this imaging workflow may be streamlined by removing such ex vivo analysis, although other complementary ex vivo techniques like high-resolution autoradiography could be added.

### Outlook

Image acquisition throughput could be increased further by adapting and/or reducing the number of imaging time points (as done in this study shortening 0…2 h dynamic to 1–2 h static scans) and by increasing the number of animals in the field of view (e.g. with a 3D-printed 4-bed hotel (inviCRO)). Additional automated analyses can be integrated into the workflow at little cost in extra time, for example kinetic modelling (with image-derived input function) or radiation dosimetry. Analysis of dual isotope images can be done practically as quickly as that of single isotope images, but the study schedule would have to factor in additional radiochemistry resources and development time. Additional valuable information might be extracted from SPECT images when replacing CT by anatomical MR images with superior soft tissue contrast, ideally in future with a mouse hotel compatible MRI setup and with simultaneous MR and nuclear image acquisitions. Finally, analysis of nuclear images of radiopharmaceuticals in clinical trials may benefit from the automated analysis procedures developed.

## Additional file

Additional file 1: Detailed descriptions of the radiolabelling procedure, image analysis, γ-counting procedure and analysis, and intersubject variability estimation. Further results of the γ-counter calibration, ex vivo/imaging comparison and intersubject variability. (DOCX 171 kb)

## Abbreviations

3Rs: Replacement, reduction and refinement
COV: Coefficient of variation
CT: Computed tomography
HPLC: High-performance liquid chromatography
ID: Injected dose
ITLC: Instant thin layer chromatography
iv: Intravenous
MIP: Maximum intensity projection
MRI: Magnetic resonance imaging
PACS: Picture Archiving and Communication System
PDX: Patient-derived xenograft
QC: Quality control
ROI: Region of interest
SCID: Severe combined immunodeficiency
SD: Standard deviation
SPECT: Single-photon emission computed tomography
SUV: Standardized uptake value
