# Supplementary material for: High-throughput high-volume nuclear imaging for preclinical in vivo compound screening§
Source: EJNMMI Res. 2017 Apr 7;7:33. doi: 10.1186/s13550-017-0281-4 (PMC5383912; doi:10.1186/s13550-017-0281-4)
Supplement: Additional file 1: — Detailed descriptions of the radiolabelling procedure, image analysis, γ-counting procedure and analysis, and intersubject variability estimation. Further results of the γ-counter calibration, ex vivo/imaging comparison and intersubject variability. (DOCX 171 kb) [file 13550_2017_281_MOESM1_ESM.docx]

Additional file 1

Supplement to: Sven Macholl, Ciara M. Finucane, Jacob Hesterman et al., **High-throughput high-volume nuclear imaging for preclinical in vivo compound screening** (EJNMMI Research, 2017).

Table of Contents

[Materials and Methods 1](#_Toc478976933)

[Detailed Radiolabelling Procedure 1](#_Toc478976934)

[SPECT/CT scanner quality control 2](#_Toc478976935)

[Image analysis 2](#_Toc478976936)

[γ−counting and analysis 3](#_Toc478976937)

[γ-counter calibration 4](#_Toc478976938)

[Intersubject variability estimation 4](#_Toc478976939)

[Results and Discussion 4](#_Toc478976940)

[Ex vivo / imaging comparison 4](#_Toc478976941)

[Intersubject variability 5](#_Toc478976942)

[References 6](#_Toc478976943)

# Materials and Methods

## Detailed Radiolabelling Procedure

Small aliquots of all 25 proteins were radiolabelled first for testing, and subsequently larger aliquots were radiolabelled for imaging. For the imaging study, usually 2 or 3 proteins were selected on any one day to be radiolabelled with a single Isolink kit vial (Paul Scherrer Institute, Switzerland) and 2.5 GBq of ^99m^Tc-pertechnetate in 0.5 ml (from the Radiopharmacy Department at St Bartholomew’s Hospital). The kit/^99m^Tc mixture was heated to 100 ˚C for 30 min, then left to cool for 10 min. Vial contents were transferred into a 1.8 ml Eppendorf tube and the pH adjusted to 7 to 7.5 with typically 130 µL hydrogen chloride solution (1 mol/L) to give the ^99m^Tc-carbonyl solution for protein labelling.

For each protein, the required amount of protein in solution (1.6 to 1.9 nmol, concentration 0.6 to 9.3 mg/mL, molecular weights 14 to 87 kDa) was transferred into a 0.5 mL Eppendorf Protein LoBind tube (Sigma-Aldrich) and 300 µL of the prepared ^99m^Tc-carbonyl solution were added. An ITLC-SG was performed for each protein with 0.1 mol/L citrate (pH=6) after 60 and after 100 min incubation at 37 ˚C. Meanwhile, two GE Healthcare NAP-5 columns (Sigma-Aldrich) were prepared with PBS supplemented with 0.1 % bovine serum albumin.

The first protein was incubated for 2 h and then purified as described below after which the purification of the second and subsequently third protein (if present) was performed. The contents of the reaction vial were transferred onto the top of the prepared NAP-5 column. The reaction vial was rinsed with 0.1 % bovine serum albumin supplemented PBS and added to the column to give a total volume of 500 µL of solution transferred onto the column. One fraction of 500 µL and another 5 fractions of 200 µL were collected. The radioactivity of each fraction was measured in a dose calibrator. The 3 fractions containing the protein peak (normally fraction numbers 3, 4 and 5) were collated. The contents of the vial with the purified product were weighed and an aliquot of fraction 6 was added to give 625 µg in total. The radiolabelled protein solution was weighed and radioactivity was measured in a dose calibrator. This solution was then split into three equal parts for injections into 3 animals.

Labelling efficiency and radiochemical purity were measured by ITLC-SG developed with 0.1 mol/L citrate (pH=6) using 1 µL of the reaction mixture. A further 5 µL were diluted with PBS to 25 µL. Of this, 20 µL were analysed by size exclusion-HPLC using a Biosep-3000 column eluted in 0.1 mol/L phosphate buffer (pH=7) at 0.5 ml/min.

## SPECT/CT scanner quality control

All manufacturer recommended tests and adjustments were performed as per the recommended schedule, and SPECT quantification calibrations with the mouse hotel setup were performed days before and after the imaging study. For the CT, a geometrical calibration was done every 4 months, and offsets and gains were calibrated weekly along with an X-ray run-up. For the SPECT camera, in addition to the quantification calibration, two measurements were done every month: a near-field flood measurement to confirm detector uniformity (employing a ^99m^Tc “point source“, with multi-pin hole apertures removed), and a spatial resolution confirmation (Jaszczak phantom with 1.0 to 1.5 mm diameter rods filled with ^99m^Tc).

## Image analysis

All image processing was performed in VivoQuant 2.0 (inviCRO) and iPACS (inviCRO). Preprocessing included a check of the SPECT/CT coregistration, upsampling and interpolation of SPECT images to the CT image voxel resolution of (0.4 mm)^3^, extraction of 3 individual mouse images from the mouse hotel image and entering subject identifiers. A script on the iPACS then generated a spreadsheet of all individual SPECT/CT images present in the database project folder allowing to enter and check all injection dose details and daily body weights. VivoQuant then expressed the SPECT image intensity in units of absolute radioactivity, in units of injected dose and as SUV. A VivoQuant script generated rotating maximum intensity projection (MIP) movies and single slice images in all 3 orientations through the tumour ROI centre. A linear colourscale was chosen for the 0.2 to 20 % ID/mL SPECT intensity range. All files were uploaded automatically to the image management database.

Tissue uptake data from SPECT images were generated via multi-atlas segmentation. This required the manual development of a reference library of ROIs, usually done in 10 to 20 animals, then building an ROI atlas, and finally applying this on all SPECT/CT images by linear- and nonlinear-registration. ROIs included tumour, muscle, liver, kidneys, heart and left ventricle.

The tumour ROI was drawn manually by 3D paint brush using the CT as reference while checking tumour boundaries against the SPECT. ROIs for the quadriceps muscle in the left leg, liver, each kidney, heart and left ventricle were defined by manually fitting ellipsoids of fixed volume to the respective organs in each CT image. ROIs for kidneys, heart, left ventricle and tumour cover the majority of each organ or tissue. ROIs in quadriceps muscle and liver only subsample those tissue types of the whole body and calculated % ID data should be ignored. In principle, whole body % ID data for these tissues can be estimated via body composition factors from literature or from this study’s dissection data, see also following section.

A master spreadsheet was generated automatically from all ROIs and their respective SPECT images listing for each ROI: volume, radioactivity, mean radioactivity concentration, radioactivity as fraction of the injected dose (% ID), % ID per ROI volume, and SUV. Furthermore, the % ID/mL ratio of tumour to selected tissues (left ventricle as proxy for blood, quadriceps muscle, each kidney) was calculated.

A Matlab script was written to read in the master spreadsheet and generate data plots by group (i.e. compound) and/or by ROI class (i.e. organ, tissue).

## γ−counting and analysis

Immediately after each last image acquisition, animals were culled by cervical dislocation and the following tissues collected: tail, urine (sample), blood (sample), brain, lungs, heart, liver (with gall bladder), spleen, stomach, intestines, kidneys (separate, with adrenal glands and fat removed), skeletal muscle (sample of one quadriceps) and tumour. Each tissue sample was weighed on a balance (Sartorius ME235P, 0.1 mg accuracy) which was interfaced to a computer allowing the push-button transfer of weight figures directly into an Excel 2010 (Microsoft) spreadsheet template, using the Accessibility Options in Windows XP and a custom-written Excel macro. That macro accepted and formatted incoming data into a table prepared with unique identifiers (compound identifier, subject identifier, tissue name). Next, radioactivities of the daily batch of tissue samples (≈100 for 2 compounds) were measured on a γ−counter LKB Wallac 1282 Compugamma within 2 hours. This produced a file with counts-per-minute data for all tissues and all compounds studied that day, decay corrected to the start of γ−counting. All new data files were added to the library of compounds studied so far. A custom-written Matlab script then imported all files and linked associated data from the different sources via unique compound and mouse identifiers. Subsequent calculations included conversion of radioactivity cpm data into absolute becquerel or curie, correction of the injected dose for residual radioactivity in the tail (injection site) and for the radiochemical purity of the particular radiopreparation, normalization of tissue activities to the corrected injected dose (% ID), taking additionally the respective tissue weight into account (% ID/g) and finally also taking the body weight into account (SUV). Tissue specific calculations were as follows.

For urine, only % ID could be calculated, and only for the urine present at the time of animal sacrifice.

For blood and muscle, the taken tissue samples were extrapolated to % ID of the whole body tissues via the factor $\frac{body\_weight \cdot body\_composition\_factor}{sample\_weight}$ with these estimated body composition factors:

1. For blood, $0.076\frac{g \text{whole blood}}{g \text{body weight}}$, based on $0.072\frac{mL \text{whole blood}}{g \text{body weight}}$ from ref.[1] and the specific gravity of human whole blood of 1.056 g/mL at 25 °C from ref. [2] assuming the value for mice is the same
2. For muscle, $0.43\frac{g \text{muscle tissue}}{g \text{body weight}}$, based on mixed-sex, adult (150 day old) rat data in ref.[3] assuming the value being similar for female SCID mice.

Data of each individual’s two kidneys were combined.

For tumour, additionally the concentration (% ID/g) ratio to selected tissues (blood, muscle, kidneys) was calculated.

Finally, all data were automatically (1) saved in a csv file, and (2) plotted in various predefined ways, e.g. for each compound (for each set of 3 animals), and for each tissue (across all compounds), with plots saved individually and as slide deck.

## γ-counter calibration

γ-counter calibration was done with five ^99m^Tc-pertechnetate samples of 18 to 46 MBq (dose calibrator Capintec CRC-15R) that were repeatedly measured on the γ-counter on days 3 to 6 after preparation. The expected activity in becquerel at each measurement was calculated by extrapolation from the corresponding dose calibrator measurement. The total of 42 γ-counter measurements spanned 40 cpm to 1.4 Mio cpm and a dead time fraction of 0.7 % to 40 %. A linear fit of the γ-counter counts-per-minute (or counts-per-second) versus the expected activities in becquerel was excellent with R^2^=0.9997.


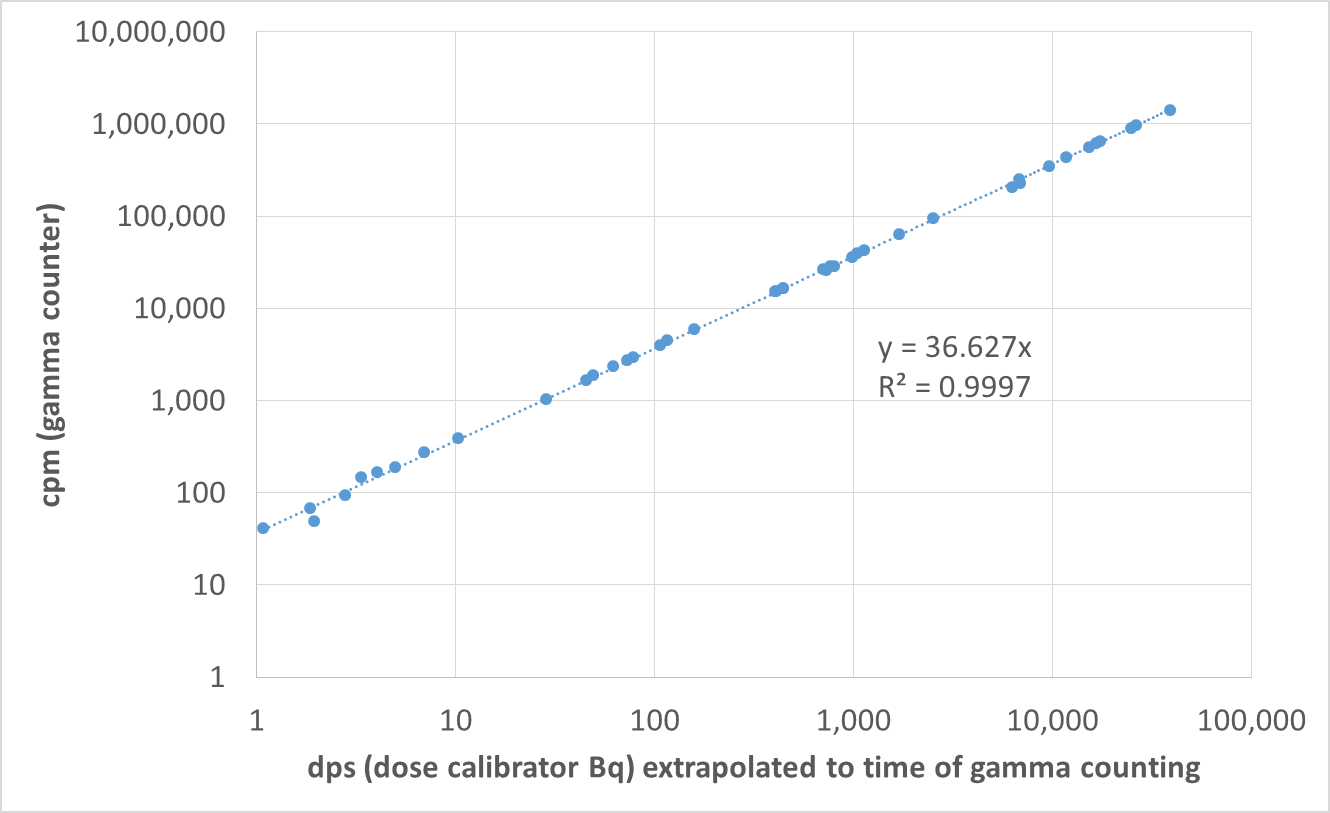


## Intersubject variability estimation

To estimate intersubject variability from SPECT/CT data, first the coefficient of variance (COV = “group standard deviation” / “group mean”) was calculated for each group of 3 mice for each uptake measure (% ID for kidneys and heart; % ID/mL and SUV for kidneys, heart, liver and tumour) for each compound and time point. Then the median was taken over all compounds and all time points, except selecting only a single frame (at ca. 1 h post injection) of each dynamic scan since the other frames’ COV data of each dynamic scan are not independent. Median values were obtained either per uptake measure/organ combination or per uptake measure across all organs.

# Results and Discussion

## Ex vivo / imaging comparison

In addition to the Bland-Altman plots presented in the paper as Figure 4, correlation plots of the same data are shown here for illustrative purposes only.


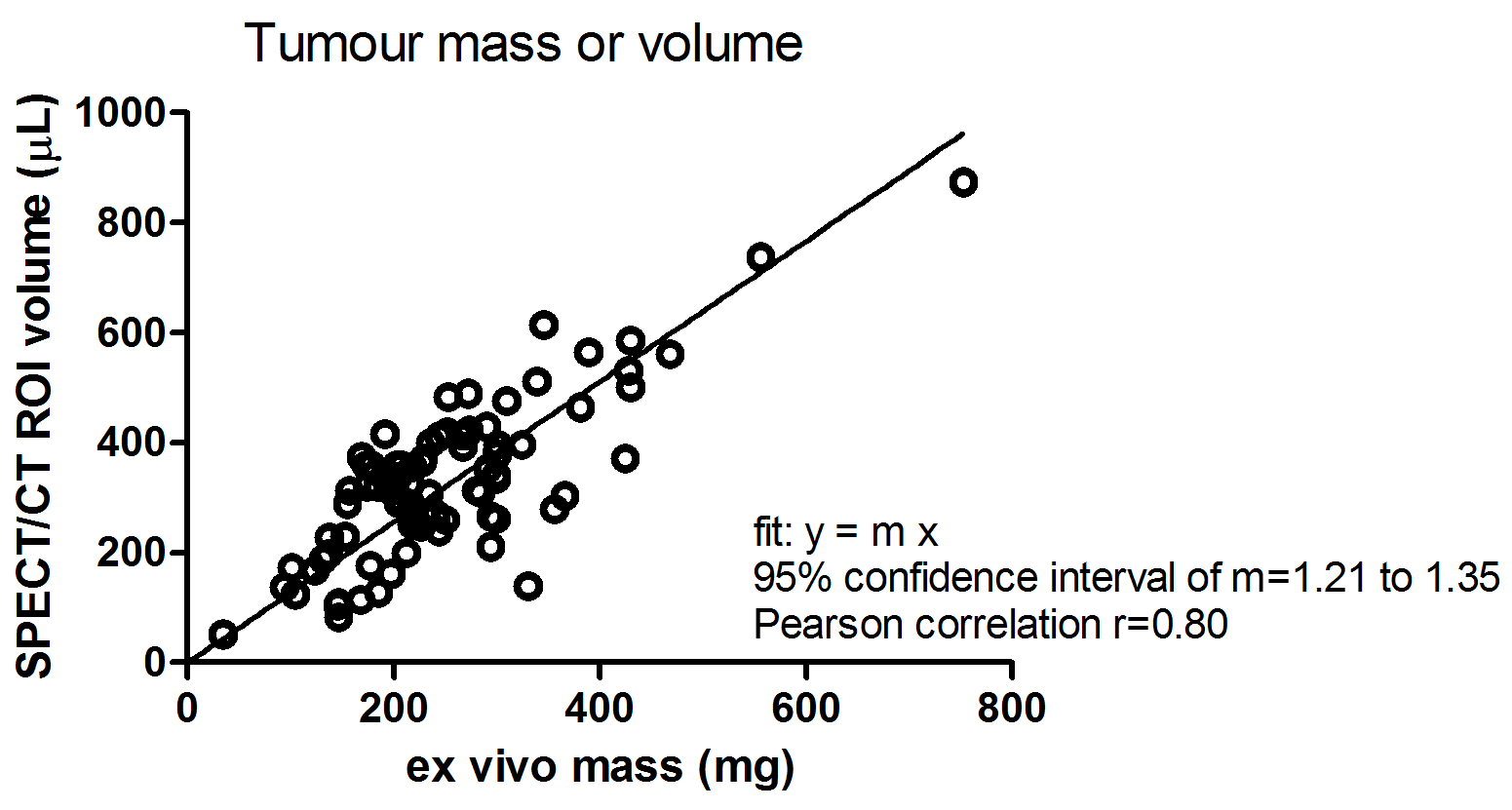


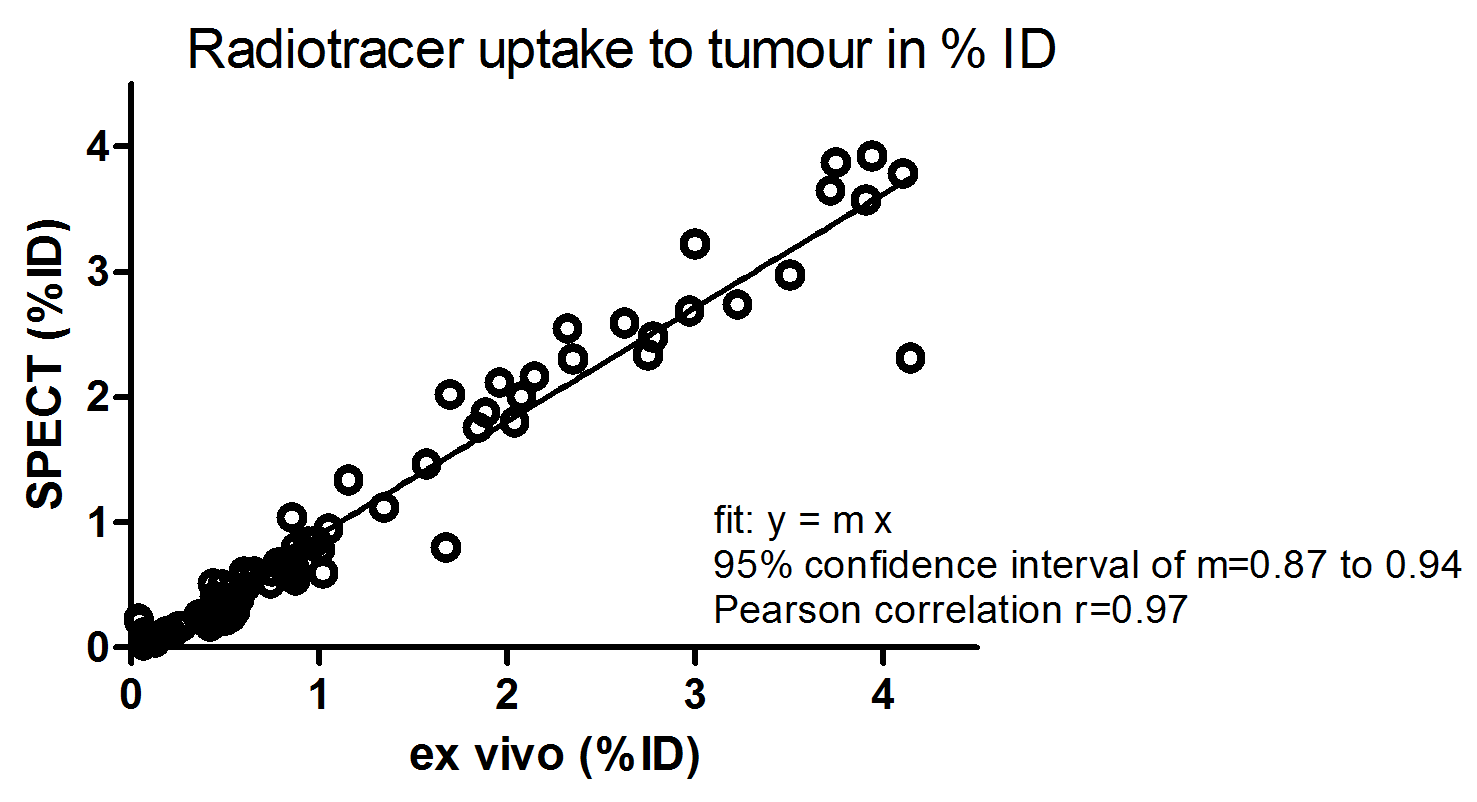


## Intersubject variability

Results: Intersubject variability estimates from SPECT/CT data are listed in Table 1 as median % COV values for each group of 3 mice across 1, 4, 24 and 48 h time points and across all compounds.

Table 1: Intersubject variability as median of “% COV” values

| Organ | % ID | % ID/mL | SUV |
| --- | --- | --- | --- |
| Liver | excluded | 9 | 7 |
| Left kidney | 10 | 12 | 10 |
| Right kidney | 11 | 11 | 13 |
| Tumour | excluded | 16 | 15 |
| Heart | 18 | 19 | 19 |
| All of the above | 13 | 13 | 13 |

Discussion: Muscle data were omitted because of generally low absolute uptake and little interest in this organ other than to eliminate compounds with a very low “tumour % ID/mL” to “muscle % ID/mL” ratio.

Left ventricle data were not considered because of a generally challenging ROI placement which increases intersubject variability substantially compared to the organs of primary interest.

Liver % ID was excluded because this was based here on a small sub-sample ROI. In principle liver % ID could be estimated from the sub-sample ROI together with the sample ROI volume, body weight and an experimental scaling factor (average liver volume per body weight in the animal population, e.g. from dissection), but this was out of scope in this study for the liver SPECT/CT data.

Tumour % ID was excluded because the tumour volume (absolute, and relative to body weight) varied substantially between animals which likely dominates intersubject variability calculated with the method used here.

# References

1. Diehl KH, Hull R, Morton D, Pfister R, Rabemampianina Y, Smith D, et al. A good practice guide to the administration of substances and removal of blood, including routes and volumes. J. Appl. Toxicol. JAT. 2001;21:15–23.

2. Trudnowski RJ, Rico RC. Specific gravity of blood and plasma at 4 and 37 degrees C. Clin. Chem. 1974;20:615–6.

3. Donaldson HH. The Rat: Data and Reference Tables [Internet]. 2nd ed. 1924. Available from: http://archive.org/stream/ratdatareference00dona/ratdatareference00dona_djvu.txt
